# Access to mental health and addiction services for youth and their families in Ontario: perspectives of parents, youth, and service providers

**DOI:** 10.1186/s13033-023-00572-z

**Published:** 2023-03-14

**Authors:** Toula Kourgiantakis, Roula Markoulakis, Eunjung Lee, Amina Hussain, Carrie Lau, Rachelle Ashcroft, Abby L. Goldstein, Sugy Kodeeswaran, Charmaine C. Williams, Anthony Levitt

**Affiliations:** 1 Factor-Inwentash Faculty of Social Work, University of Toronto, 246 Bloor Street West, Toronto, ON M5S 1V4 Canada; 2 Family Navigation Project, Sunnybrook Research Institute, Toronto, ON Canada; 3 Department of Applied Psychology and Human Development, Ontario Institute for Studies in Education, Toronto, ON Canada; 4 Family Navigation Project, Sunnybrook Health Sciences Centre, Toronto, ON Canada

## Abstract

**Background:**

Canadian youth (aged 16–24) have the highest rates of mental health and addiction concerns across all age groups and the most unmet health care needs. There are many structural barriers that contribute to the unmet mental health care needs of youth including lack of available and appropriate services, high costs, long wait times, fragmented and siloed services, lack of smooth transition between child and adult services, stigma, racism, and discrimination, as well as lack of culturally appropriate treatments. Levesque et al. (2013) developed a framework to better understand health care access and this framework conceptualizes accessibility across five dimensions: (1) approachability, (2) availability, (3) affordability, (4) appropriateness, and (5) acceptability. The purpose of this study was to explore access to addiction and mental health services for youth in Ontario, Canada from the perspectives of youth, parents, and service providers.

**Methods:**

This qualitative study was a university-community partnership exploring the experiences of youth with mental health concerns and their families from the perspectives of youth, caregivers, and service providers. We conducted semi-structured interviews and used thematic analysis to analyze data.

**Results:**

The study involved 25 participants (n = 11 parents, n = 4 youth, n = 10 service providers). We identified six themes related to structural barriers impacting access to youth mental health and services: (1) “The biggest barrier in accessing mental health support is where to look,” (2) “There’s always going to be a waitlist,” (3) “I have to have money to be healthy,” (4) “They weren’t really listening to my issues,” (5) “Having more of a welcoming and inclusive system,” and (6) “Health laws aren’t doing what they need to do.”

**Conclusion:**

Our study identified five structural barriers that map onto the Levesque et al. healthcare access conceptual framework and a sixth structural barrier that is not adequately captured by this model which focuses on policies, procedures, and laws. The findings have implications for policies and service provisions, and underline the urgent need for a mental health strategy that will increase access to care, improve mental health in youth, decrease burden on parents, and reduce inequities in mental health policies and services.

## Introduction

Canadian youth aged 16–24 have the highest rates of mental health and addiction concerns across all age groups [1] with increases in prevalence over the last decade [2]. More than 20% of Canadian youth have mental health concerns, 12% have substance use concerns [1], 5% have low to severe problem gambling, and 12.5% have a video gaming problem [3]. While the rates of mental health and addiction concerns are alarmingly high, youth have the most unmet mental health care needs in Canada [4, 5] with more than 75% not receiving the type of specialized mental health services needed [6]. Across all age groups, youth are least likely to seek help [4], and of those who receive services for mental health and addiction concerns, approximately 52% drop out of treatment [7]. There are many barriers and gaps that contribute to the unmet mental health care needs of youth including lack of available and appropriate services; high costs [8]; long wait times [9]; crisis driven services; fragmented and siloed services; lack of continuity or smooth transition between child and adult mental health services [7, 10]; stigma, racism, and discrimination; and lack of culturally appropriate treatment and care.[8, 11, 12] Moreover, there are increased structural barriers accessing mental health care for youth who are part of marginalized groups [11, 13].

Parents or caregivers play an important role helping youth seek mental health treatment [14]. However, the lack of services and unclear pathways to youth-specific mental health care treatment creates tremendous burden and ample confusion for parents. [5, 8, 15, 16] More recently, some agencies and hospitals have started developing navigation services to assist youth and their families seeking mental health care as this can mitigate some of the barriers to service access. Unfortunately, some families do not connect with navigation services and navigation services are not available in all agencies and jurisdictions [15].

There are several frameworks that have been developed to better understand access to healthcare. One of the most comprehensive [17] is an access framework developed by Levesque et al. [18] which defines access to health care as “the opportunity to reach and obtain appropriate health care services in situations of perceived need for care” ([18], p4). Levesque et al. [18] conceptualize accessibility across the following five dimensions: (1) approachability, (2) availability and accommodation, (3) affordability, (4) appropriateness and adequacy, and (5) acceptability. These dimensions also consider socioeconomic determinants and an individual’s abilities to perceive, seek, reach, pay, and engage with services. Approachability includes how effectively services can be reached by service users, and the extent to which services provide outreach and transparent information. Availability and accommodation reflect the ability of services to be reached in a timely and effective manner. This includes the physical and geographical accessibility of services, whether services are provided by telephone or virtually, the length of time it takes to access care, and the hours of operation. Affordability refers to direct and indirect costs. An organization’s service fees and treatment expenses, as well as their ability to cover insured or uninsured clients, impact affordability. Indirect fees can also include those related to attending appointments such as taking unpaid time off work, transportation costs, and childcare. Appropriateness and adequacy refer to the fit between the needs of service users and the actual services provided. This includes characteristics of service providers, such as competence, qualifications, attitudes, and ability to engage with clients. The final dimension is acceptability which refers to sociocultural factors that influence how appropriate the services are to meet the diverse needs of individuals, families, and communities [17, 18].

A review of studies examining parent perceptions of barriers and facilitators to child and adolescent mental health treatment found that parents perceive structural barriers as the most relevant and cumbersome access barriers [19]. Structural barriers are defined as “factors and practices rooted in social, political, legal, and service systems” ([20], p55) “that systematically hinder access for certain groups of people” ([21], p215). In a review on challenges and barriers to mental health care systems, Carbonell et al. [22] define structural barriers as “difficulties deriving from institutional policies and procedures that restrict the rights and opportunities of people with mental illness and their families” ([22], p1372) The review found that structural barriers were linked with poor public health policies, underfunded mental health care systems, treatment gaps, poor planning, and low priority of mental health by governments.

In Canada, the health care system is governed by the Canada Health Act [23] which has a primary objective to “protect, promote and restore the physical and mental well-being of residents of Canada and to facilitate reasonable access to health services without financial or other barriers” [23]. Administration and service delivery are highly decentralized across the provinces and territories and other key features of Canada’s health care system include comprehensiveness—defined as medically necessary services; universality—which stipulates that all insured residents can have uniform health services; portability—which refers to coverage across Canadian jurisdictions; and accessibility—which is to enhance reasonable access to services [24]. Ontario is the most populated province of Canada [25] and it uses Community Health Centres and Family Health Teams as the primary form of interprofessional primary health care [24, 26]. While family physicians are usually the first point of contact for publicly funded mental health services [28], many do not have the resources or supports to meet the growing mental health care needs [27]. According to Miller et al. [28], family physicians feel “lost” trying to navigate the complex and fragmented mental health care system in Canada for youth especially when they cannot access specialized care. Ashcroft et al. [27], examined how policies in Ontario influence mental health care in Family Health Teams and found that there is variation and inequity in the delivery of mental health services attributed to structural factors and lack of strategic direction.

The purpose of this study is to explore access to mental health and addiction services for youth aged 16–24. It is part of a larger study examining facilitators and barriers for families seeking mental health services for youth in Ontario, Canada. We have published a paper from the larger study that reports on the inequities in mental health care specifically linked to COVID-19 pandemic measures from the perspectives of youth, families, and service providers [8]. For the present study, we examined the following research question: How do youth, parents, and service providers describe access to youth mental health and addiction treatment and services?

## Methods

### Design and setting

For this research study, we selected a descriptive qualitative research design as it is an appropriate approach to facilitate the exploration of participants’ experiences and perspectives on youth mental health and addiction treatment and services [29]. A collaborative research approach informed this study, where knowledge is constructed by researchers and community partners [30]. This university-community partnership emphasizes principles of recovery-oriented care and service users’ lived experiences in informing changes and improvements to service provisions [30, 31]. The partners on this study were the Family Navigation Project (FNP) and the University of Toronto. The FNP provides navigation services, connecting youth and families to needed mental health and addiction services. Navigators work with about 750 families each year, linking them to more than 1100 service providers and programs across Ontario. FNP was created by families, for families, and is currently the largest provider of mental health navigation services in Canada [32]. This study received approval from the research ethics boards at the University of Toronto and Sunnybrook Health Sciences Centre.

### Study context

This study unexpectedly coincided with the COVID-19 pandemic. On March 17, 2020, the provincial government declared a state of emergency [33] and both federal and provincial governments began implementing lockdown measures that involved travel restrictions, border closures, closures of schools and businesses, and suspension of nonessential health and public services [34]. Data collection for this study took place in spring and summer 2021 which was the third wave of the pandemic in Ontario, Canada [35]. Each wave of the pandemic has been described as having specific characteristics described as a “health footprint.” The third wave of the pandemic was characterized by the impact of interrupted care on chronic conditions [36].

### Sample and recruitment

We used a purposeful sampling strategy [37] to recruit service users and service providers who are knowledgeable on access to mental health care through professional and lived experiences. Service providers working in a variety of settings (e.g., hospitals, community agencies, and private practice) were eligible to participate in the study if they had previously worked with youth or families connected with the FNP. All navigators at the FNP were eligible for participation, based on their experiences working directly with youth and their families. Service users included youth aged 16–24 and parents/caregivers of youth aged 16–24 who had previously received navigation services from the FNP. Researchers sent recruitment flyers to the FNP and FNP staff sent the flyers to service users and providers. The recruitment flyer included a link to a consent form that provided a description of the study. A research assistant from the University of Toronto contacted participants after they provided consent to participate in the study. Participants received an honorarium in the form of a $25 gift card for their time.

### Data collection

The research team collected data from March to July 2021 through semi-structured interviews conducted by a research assistant (AH) and/or the principal investigator (PI: TK). Interviews were approximately 60 minutes in length and took place by telephone or virtually using an online video conferencing platform. Prior to the interview, participants completed a brief online demographic questionnaire that collected the following information: age, gender, role of caregiver (e.g., father, mother, grandparent), job title for service providers, and living arrangements for youth (e.g., living with family or independently). We developed a semi-structured interviewing guide with several open-ended questions [38] that focused on mental health and addiction service access for youth and families not specifically related to the pandemic. However, a few questions were added that asked about mental health care access in the context of the pandemic, and findings specifically pertaining to the pandemic are reported elsewhere [8]. Questions were grouped in the following categories: (1) youth, parent/caregiver, and service provider experiences; (2) mental health and addiction services used currently or in the past; (3) identified mental health and/or addiction-related concerns that prompted request for navigation; (4) barriers and facilitators for accessing mental health and addiction services; (5) the role of families and the impact of family involvement; and 6) youth and parent/caregiver experiences of discrimination, racism, and stigma. All interviews were audio recorded, transcribed, and participants were de-identified using ID codes. The raw data were only accessible to the PI and research assistants.

### Data analysis

Data were analyzed using thematic analysis which is a six-stage qualitative method for identifying and analyzing patterns and themes within data [39]. Data analysis was completed by the PI, research coordinator, and two research assistants, who used the software Dedoose to organize and synthesize the data. The four research team members reviewed transcripts in detail, prepared memos for points of discussion, and engaged in discussion to achieve familiarity with the data. Research team members generated codes, following a recursive process of identifying possible codes, developing definitions for these codes, returning to interview transcripts, and further revising the set of codes as necessary [39]. We maintained a codebook with code names and definitions and met weekly to debrief coding. After extensive review of the codes, we identified six overarching themes.

This study used several strategies to enhance credibility, dependability, confirmability, and transferability [40]. Procedures included triangulation of data sources, researcher triangulation, prolonged engagement with the data, documenting observations during interviews using field notes, memoing throughout the data collection and analysis stages, and thick description of participant quotes. Moreover, we minimized researcher bias by writing reflexive memos and conducted regular research team meetings where we debriefed and wrote thorough notes that captured our reflexive discussions.

## Results

The study involved a total of 25 participants (n = 15 service users, n = 10 service providers). Among the service users, 11 participants were parents and four were youth. Most of the parents were mothers (n = 10) and one participant was a father. Three parents were supporting a daughter with mental health and/or addiction concerns and eight were supporting a son. None of the youth participants were connected to the parent participants in the study. Table 1 provides more information about participant characteristics.

**Table 1:** Participant characteristics

Characteristics n (%)	Parents (n = 11)	Youth (n = 4)	Service providers (n = 10)
Gender			
Male	1 (9%)	–	1 (10%)
Female	10 (91%)	4 (100%)	8 (80%)
Did not identify	–	–	1 (10%)
Age (mean)	57	21	–
Self-identified ethnoracial identity			
Black	–	1 (25%)	–
South Asian	1 (9%)	1 (25%)	1 (10%)
East Asian	2 (18%)	1 (25%)	–
White	7 (64%)	1 (25%)	8 (80%)
Mixed race/biracial	1 (9%)	–	1 (10%)
Parent role			
Mother	10 (91%)	–	–
Father	1 (9%)	–	–
Youth supported by parents/caregivers			
Son	8 (73%)	–	–
Daughter	3 (27%)	–	–
Highest level of ducation			
High school	–	3 (75%)	–
College diploma/undergraduate university	8 (73%)	1 (25%)	–
Graduate degree university	3 (27%)	–	10 (100%)
Educational degree			
Master of Social Work	–	–	8 (80%)
Master of Education	–	–	2 (20%)
Identified role for service providers			
Social worker	–	–	1 (10%)
Crisis worker	–	–	1 (10%)
Clinician navigator	–	–	7 (70%)
Did not identify	–	–	1 (10%)
Practice setting			
Private practice	–	–	–
Hospital	–	–	6 (60%)
Both hospital and private practice	–	–	3 (30%)
Post-secondary institution	–	–	1 (10%)
Mean years of practice experience	–	–	10.6
Type of concerns^a^			
Mental health	4 (36%)	3 (75%)	–
Substance use/behavioural addictions	1 (9%)	–	–
Concurrent disorders	6 (55%)	1 (25%)	–

^a^Type of concerns for the parent/caregiver group refers to the self-reported mental health and/or addiction concerns of the youth they are supporting

We identified six themes that described structural barriers impacting access to youth mental health and addiction treatment and services: (1) “The biggest barrier in accessing mental health support is where to look,” (2) “There’s always going to be a waitlist,” (3) “I have to have money to be healthy,” (4) “They weren’t really listening to my issues,” (5) “Having more of a welcoming and inclusive system,” and (6) “Health laws aren’t doing what they need to do.”

### “The biggest barrier in accessing mental health support is where to look”

Most participants in our study described how services were not approachable and they underlined that there are fragmented services and unclear pathways to youth mental health care. A youth stated that “the biggest barrier in accessing mental health support is where to look” (Y15) and a parent underlined that “accessing services is hard. It’s hard to know where to start. It’s hard to know where to go” (P5). Another parent noted that “family doctors don’t even know where to send you” (P7). One of the service providers explained,

I think one of the gaps or barriers is just how segmented the system is…they're very, very segmented or separated, it's hard to access, there's long wait times, or it's not always a clear pathway to make a referral. There's often lots of hoops and appointments and people to meet. And I think that's a huge barrier for young people who have a hard time accessing services and then to have to go through all of these different pathways. (SP16)

Another access barrier described by participants is inadequate outreach and information about available mental health and addiction services for youth and their families. A service provider stated, “our system is not doing enough to make people aware on where to access help for what reasons” (SP17). Another service provider added, “we just need to find a way to kind of spread the word of what's available and how to access it” (SP19). Service providers also underlined that some populations are likely more affected by the lack of information about available services.

I think one of the challenges is that we don't outreach to different communities. Families that connect with us do it by word of mouth or professionals in the field. So, I think there's a lot of gaps in populations that aren't probably accessing our services because how would they know about us? Because we haven't shared it to everybody. (SP2)

An additional barrier affecting access to youth mental health care described by participants is not having a smooth transition from children’s mental health to adult services. A service provider explained,

Some of the young people that I work with…notice the huge transition in accessing care at [name of children’s service] for example, to then being referred to an adult specialist, and that's a really hard transition, and there isn't, I think, enough supports in place for young people making that transition…I really want to emphasize that. (SP16)

Most participants noted that navigation services helped cope with the barriers related to the lack of approachability of services. Navigators helped provide information about youth mental health and addiction services and they contacted services for parents and youth which reduced access barriers to some extent. A parent explained perceiving that navigators receive more immediate responses from service providers than parents:

I was looking for information and calling up and people were just ignoring me, because I guess I am a parent... [the navigator] got a response right away…it makes a huge difference…she initiated the call to the agency for me, and she made all the connections. (P24)

A youth also noted how beneficial it was to have a navigator assisting with access to appropriate services: “the [navigator] would try to set up a phone call, like at least once a week, to kind of talk about different resources and kind of provide me with different options…about different services that could help me out” (Y21).

### “There’s always going to be a waitlist”

All participants identified barriers related to the availability of services and this was associated with difficulties reaching the service, wait lists, as well as closed or altered services. For example, all three participant groups stated that even when they finally identify a potential mental health service, there are barriers in reaching the service provider particularly for youth in distress. One service provider explained:

The biggest barrier would be that youth are expected to call back places themselves, and when youth are unwell, they're not able to do that. So, services end up being cancelled because the youth didn't get the message or didn't return the message or didn't show up for the appointment because the parent didn't know about the appointment and couldn't remind them or take them. That's a huge issue. I talk about this all the time with parents, it's so frustrating. So frustrating (SP1).

A youth described the search for services as “emotionally exhausting” and explained that this impacts mental health:

I have a very difficult time just getting on the computer, narrowing it down…then you have to call, and then you have to go on all these waiting lists, and sometimes they'll hold you for hours to just speak with someone. And it's very discouraging because I have addiction and I have depression and I have anxiety, so…me already getting on the phone, it's already triggered my anxiety and my depression (Y23).

Wait lists were also a barrier described by participants across the three groups and they underlined that pandemic measures amplified wait times. Moreover, service providers explained that the wait lists were longer for youth requiring specialized or culturally adapted services.

I would say, public, free services for families, you can always expect a waitlist…anything that starts to get more specialized has longer waitlists…there's always going to be a waitlist for a psychiatrist. And then if you need, also, that psychiatrist to have specialty in ASD or assessing something more specific, then that has a waitlist…there's different levels of waitlists too (SP19).

Youth noted that being on wait lists can be discouraging and result in disengagement. “Just being told I was going to be waitlisted…makes me not want to do it…having to wait 8 months makes me realize…in 8 months, am I gonna still be in tuned with how I'm feeling right now?” (Y15). Another youth expressed not understanding the wait times:

Everywhere I go, it's six months' waiting. So, I've talked to lots of places but lots of places have waiting lists, and I have addiction and depression, I don't know how people can put you on a waiting list…They'll tell you that you have to call back in six months because they're too full and then it's just like, discouraging because it's not like you do it for no reason…You need help now and you're not going to get help for six months…I think that that is a ridiculous time to wait. (Y23)

Another service provider described the challenges of finding available spots in residential or intensive treatment programs especially due to COVID-19 pandemic restrictions that resulted in reduced available spots, unexpected closures, and changes in format of services from in person to virtual:

Residential treatment is a huge challenge. And I think outpatient…more intensive services because of a lot of them don't have the ability to take people in person. And so, I think that's not necessarily a wait time challenge but a service challenge, like not being able to meet the needs of people who need that intensive service…They just had to stop their service, or offer something virtually, so I think that's another gap for people that need more intensive, there's not a lot of options. So, they don't even bother going on the waitlist, they just know that their needs won't be getting met. (SP2)

A parent described the waitlist for residential treatment for a youth with substance use concerns: “There are over 200 youth on the waitlist…substance use disorder is a potentially terminal illness, kids can't be waiting 14 months for residential treatment. I mean, how are you going to keep them alive in the meanwhile?” (P10). Many parents described the importance of peer support services, especially when professional services are unavailable. One parent underlined,

It's a group of parents who have kids who are struggling with all kinds of issues, we meet once a week, we talk about how our week has been…Parents who have kind of trained to become leaders, not professionally, just by being in the group, sit down and work with you on a plan to address one major issue that's causing you concern that week, or something that you want to work on, and you work with practical things you can do that next week to get through the week, or get through the crisis…Any one of the leaders, you can call them anytime, night or day, and they'll help you out if you're in a crisis…I don't know how I would have survived without this group of parents. (P20)

### “I have to have money to be healthy”

Most participants in all three groups highlighted the issue of affordability related to youth mental health and addiction services. There was a recurring theme of public versus private services and the inequitable access to care. Many of the service providers noted that when youth and parents have insurance or are able to pay for private services, they will usually recommend this option because the wait lists are shorter. According to one service provider, “I can speak to the difference between private care and public care…The more resourced you are, the easier it is to access services” (SP8). A parent spoke about the financial burden caused by unavailable public services and the need to pay for private: “I feel that we would not be in the hole that we are in now, if I were able to afford therapy…that's the number one barrier, is not being able to afford the private therapy that's available” (P9). Another parent shared concerns about the financial strain:

It's an extreme burden for parents to carry, not only are they carrying the burden of having somebody under their roof that is struggling, and they, in turn, are struggling. But then to have to worry about, you know, how much is their health worth to you? Is it worth selling the home? (P20)

A youth also expressed frustration about the need for financial resources to address mental health concerns:

I find it absolutely ridiculous that you have to pay somebody, like I have to be able to have money to be healthy…and then if I can't—when you have mental health problems, sometimes you can't get a job…until my mental state is fixed, I will not be able to get a job. So, then, what…? You're just going to have all these people that are depressed and you're going to be like, f**k you because you don't have money? (Y23).

Service providers explained that public (cost free) specialized services are even more difficult to access through publicly funded centres: “Specific diagnoses…like OCD… it needs a specific treatment…When a family is looking for support and the only option is something that costs a lot of money, it is not helpful to lots of families who can't afford that” (SP19). Parents and service providers underlined that public residential treatment options were very limited with very long wait lists as noted by this parent: “You can skip the queue…and get into [residential treatment centre] in three weeks. If you don't have the money, you're waiting 14 months” (P10). Service providers also explained that during the pandemic there were greater disparities between families who had greater financial resources than those who did not. “When you're working with clients who are more marginalized or vulnerable or finances are a major issue, then of course, there's going to be tons of barriers that exist for them. And then that exacerbates the mental health or the addictions issue that they're struggling with” (SP8). This service provider also described the inequitable access to mental health care for international students, “I work with a lot of international students…finding them resources when they're not covered by OHIP…It's literally impossible…sometimes they have insurance, but these are students who are paying three or four times the amount that domestic students are paying” (SP8).

### “They weren’t really listening to my issues”

Many participants stated that there were challenges finding appropriate and adequate services that fit with the needs of the youth or family. One youth described, “it wasn't really helpful…I have gone to a lot of services, and I feel like I’ve been hearing the same things that they were saying, like it was protocol, like they weren't really listening to my issues” (Y21). A service provider explained that sometimes youth are hospitalized due to limited outpatient options:

There continues to be a gap in the system around something more intense for youth. A lot of youth don't necessarily need to be in-patient in psychiatry, in a hospital, but there's not a lot of other options, or something specialized. So, when they're looking at outpatient treatment, or like a day treatment, there's not something that is specialized for their mental health…residential treatment for mental health is really very limited as well. (SP2)

While some participants described mental health care services using a “one size fits all” approach, others described service provisions having a narrow focus making these services inadequate for some youth. A parent explained,

If he gets the treatment for substance use disorder, you usually don't get the treatment for the co-occurring disorders. Like, it's a huge problem. Like, we've got to get rid of those silos…They need to break down all the silos, so kids get assessed for everything, you know, as soon as possible, and they get a treatment plan. (SP10)

Participants also discussed the inappropriate modalities of treatment and the overfocus on individual approaches to care versus family-centred approaches. According to one service provider,

Family members... We're often thinking about strategic ways about how they can be involved more in their care, and ways that their youth are okay with...The way the system is kind of set up, it's not naturally having family involvement, at least not the medical system…Therapy is not set up to be family-involved, like psychiatry is not necessarily set up that way. And then there's the youth groups and programs that are youth centered, but not for families. (SP2)

The format of service delivery was raised by many participants as many of the services were not offering in-person services due to pandemic lockdowns and restrictions. Many participants felt that virtual mental health care was inappropriate for some youth:

All of it is virtual or by technology…None of it is in person and so that doesn't necessarily work for all families…I have families of youth with autism…Some youth that are developmentally delayed…or if somebody is experiencing paranoia or psychosis, they may not be onboard with it. (SP12)

Parents and youth described some experiences with poor quality mental health care. One parent stated, “I have witnessed treatment of mental health patients, it's not impressive, very stigmatized…the care that's provided in a facility funded by the government is really subpar” (P22). Another parent described an interaction with a service provider:

Was upsetting…he said to me…why isn't your son out working? I'm like, yeah, why isn't he? Like, you tell me! He has mental health; he's smoking pot all day…help me. And then, he was very accusatory and very rude. Like, I had 5 minutes and he just didn't want me there. (P25)

Another parent underlined that it is difficult trusting service providers due to the power they hold and how that can lead to use of coercive measures:

They coaxed him back in…And tied him up…Think of how bad I felt. So, this was the beginning of all this crap…There is no services, you can't trust people. You say the wrong thing at the wrong time, they're gonna tie you up (P7).

### “Having more of a welcoming and inclusive system”

Participants from all three groups described concerns and gaps in the acceptability of services and the need for a more “welcoming and inclusive system” (SP16). A service provider stated that “the way in which it's set up is not necessarily client-friendly for all people. I think there's certain power dynamics…and so, it may deter youth from wanting to engage with the services” (SP2). One parent described “stigma against families” and added that the lack of training for service providers contributes to more judgmental approaches:

There's stigma with service providers. You know, the most dangerous stigma is we haven't properly educated doctors…they view it as a choice and you're a bad person, and if you're using illegal drugs, you're a criminal, so the right thing for you is to go to jail…they don’t need your judgment. (P10)

Another service provider emphasized that “social workers are not being trained with AOP^1^ or cultural attunement” noting that “there are tons of barriers that exist when we're working with marginalized, racialized clients. For example, it's very difficult for me to find racialized service providers, so that's a huge barrier” (SP8). Another service provider added that there are challenges in finding culturally responsive services in public institutions: “The private sector does a better job at giving people more access to what they're looking for…If you want a Black therapist, these are the Black therapists. With public services, you get what you get, and you don't get upset” (SP14). Most service providers stated that finding appropriate services for marginalized youth was even more difficult during the pandemic due to lockdowns: “I think the LGBT community is really struggling right now…really isolated…They're not getting the access to that kind of support” (SP14). A parent also underlined the importance of inclusive services for LGBT youth: “She's gay. She had preferences for services where she knew…that she would not be an exceptionality in that case” (P9).

A youth described the family’s experiences with racism and how this contributed to mistrust of services and “family stigma” related to mental health. The youth explained that mental health is not discussed openly in their family: “Being in a Black home…it's not something that's spoken about…” The youth noted that it would be helpful if service providers would include families in the youth’s treatment and stated that service providers “didn’t know the struggles that [her parents] went through, just because they weren’t the same cultural background” (Y13). The youth expressed that giving her parents psychoeducation about mental health would be beneficial: “I really feel like them getting more knowledge on what it is, and not seeing it in such a bad light would help a lot” (Y13). Another youth stated that she wanted her parents to receive more information on mental health so they could better support her, but she explained that “my language is not offered in the general translations…so I couldn’t ever give my mom or my father a blurb of mental health because it’s just not available” (Y15).

### “Health laws aren’t doing what they need to do”

There were many concerns raised about policies, procedures, and laws impacting access to mental health care for youth and parents. Service providers and parents described concerns about confidentiality and consent laws including age of consent, consent to share information or involve families, and consent to treatment. A parent stated, “I found it really challenging…in the last year, now that he's 18…that age where all of a sudden, he needs to give consent for me to help him” (P6). Another parent added, “They're totally dependent on us, and we're not allowed to be a part of their journey or a part of their care or treatment” (P22). Most service providers in this study underlined the importance of involving families in youth mental health treatment and noted that there are challenges with the current policies and service design: “The way the system is kind of set up, it's not naturally having family involvement, at least not the medical system… we're often thinking about strategic ways about how they can be involved more in their care” (SP2). One service provider recognized that these policies and laws around consent create barriers for families.

A huge barrier that families express is around consent and confidentiality, and they appreciate that it's there for a reason, that their youth do not have to involve them in care, but it can be really challenging for family members when they don't know what's happening…They often feel really at a loss about what to do, especially if their youth won't give them consent to speak to any provider or get them any help. (SP2)

Another service provider explained that they try to reduce this barrier by coaching parents to talk to their youth about providing consent to involve them in treatment:

In terms of consent…I think it's also how we educate and facilitate consent, right? We'll coach parents…to ask them if they've asked for consent…When I work with families, I tell them that I'll work with the youth, and then I encourage consent…There's a lot more education and it's really done in a way of I pursue consent and educate around consent, rather than…there's nothing I can do…I think that there's a family-centered approach to consent as well. (SP14)

A parent described what they perceive to be the role of physicians and what would be more helpful for parents:

Doctors aren't lawyers, and they don't have a good education on addiction, which is, like, unbelievable, but it's the truth. So, they don't understand how the health laws should be applied…The health laws aren't doing what they need to do, we need physicians to advocate for the changes that will save the lives of their patients…And you know, they've just been taught…autonomy. So, that [youth] has autonomy, they don't want to stop using, that's their choice, and so there's nothing I can do, and it's like, no it’s your job to protect them from self-harming to death. (P10)

Some participants described institutional policies that impede family involvement and do not train service providers on how to use family-centred approaches:

I think at the agency or service level, it's just not having enough resources...they might need that support in terms of understanding how the family can be involved…if there are barriers with people working and not being able to support, like I just don't think there's enough resources. Our program, in our hospital, for example, is able to provide that support to families…I think resources is a big one…lack of time. (SP16)

Another service provider added,

Historically, families have been excluded, to a large extent, from the treatment process of their youth. Whether it was an issue of consent or it was sort of blaming, and identifying the individual as angry, not engaged, and not willing. I think there has been a very difficult relationship historically with families, and I think we've done a lot of work to become more family-centred, even in terms of, there are different treatment approaches or family counselling approaches that are about bringing the family into the treatment team, becoming a part of that treatment team. (SP14)

## Discussion

This study provided key insights into youth mental health and addiction treatment and service access from the perspectives of youth, parents, and service providers. Our study identified five structural barriers that map onto the conceptual framework for access to healthcare by Levesque et al. [18] including approachability, availability, affordability, appropriateness, and acceptability. We also identified a sixth structural barrier impeding access to mental health and addiction care that is not adequately captured by the Levesque model [18] which focuses on policies, procedures, and laws.

Approachability refers to how visible and identifiable services are to youth with mental health care needs. Information about available services, outreach activities, and transparency can make services more approachable [18]. Consistent with previous studies, our findings showed that youth, caregivers, and service providers described services as fragmented, with unclear pathways and gaps in transitions between child and adult mental health care [7]. For example, a study examining the experiences of youth transitioning from child to adult mental health care in Ontario found that youth experienced a lack of information about service pathways [41]. A recent survey of Ontario students in grades 7–12 found that 42% had concerns around their mental health in the last year and would have wanted to talk to someone but did not know where to go to seek support [3]. While these have been longstanding issues in youth mental health care, services have been even less approachable for youth and their families during the pandemic as many services closed or reduced service delivery making it more unclear for youth and families to know where to get help.

Saunders et al. [42] found a huge shift in the modality of mental health services for children and adolescents for the first year of the pandemic (i.e., March 2020 – February 2021) with three-fourths of outpatient mental health services delivered virtually. This rate was higher for youth more than other age groups and it was also higher in Ontario more than other jurisdictions. The authors note that in Ontario physicians received remuneration for delivery of virtual care and in jurisdictions where remuneration was lower, the uptake of virtual care was not as high. Prior to the pandemic, there were calls to address the youth mental health crisis in Canada [2, 43] and mental health concerns in youth have increased and worsened since the start of the pandemic [44]. Robillard et al. [45] found that there has been an increase in the number of Canadians screening positive for mental disorders with no history of mental health concerns. Kourgiantakis et al. [8], found that youth mental health concerns have increased, while Ontario mental health services decreased during the pandemic. Some studies have shown declines in mental health service utilization during the pandemic even though there are increased mental health care needs. There is a need to better understand the effectiveness of virtual mental health care because most outpatient services have had significant changes in their delivery as they shifted from in person to virtual [45, 46]. According to Vaillancourt et al. [43] the decline in mental health visits may be a result of changes in delivery of services during the pandemic by family physicians and school closures with both being a first point of contact for children and youth with mental health concerns. School closures in Ontario were the longest of any province or territory in Canada. Youth were disconnected from some of the settings where service providers are perceived as most approachable and youth and families were not given adequate information about available community mental health services during the pandemic [47, 48]. Vaillancourt et al. [43] report that school closures and unavailable primary care services resulted in increased visits to emergency with much higher rates of admissions to children’s hospitals in Ontario.

Our study also showed that mental health services were not available and wait lists were the most frequently identified barrier which is in line with the findings of previous studies [6, 8, 9, 14]. Kowalewski et al. [9] reported that wait times in Ontario child and adolescent mental health services (CAMHS) ranged from nine months for children with high priority needs up to one year for children deemed low priority. Fante-Coleman and Jackson-Best [11] found that Black youth have wait times that are almost double that of white youth with reduced access to family physicians. Schraeder and Reid [14] explain that not having standards for acceptable wait times does not hold CAMHS agencies and funders accountable for timely service delivery. Cairney et al. [10] found a misalignment between youth mental health care needs and availability of services in Ontario:

Regions with higher need, as demonstrated by higher rates of substance use, neonatal abstinence syndrome, hospital admissions, emergency department visits, suicide, and behavioural issues, are also areas where there are fewer outpatient services and resources, the longest wait times and the lowest rates of mental health visits by all physician types.[10 p3]

Many of our participants discussed the cost of youth mental health care services despite having a health care system in Canada that identifies universality and accessibility as two key criteria. Affordability is a structural barrier identified in previous research studies in Canada [11, 49]. In our study, participants described the lack of services available through the public health care system (especially specialized services) and they also noted that this creates inequities in who receives quality mental health care. Studies have shown that due to wait lists and unavailable services, youth and families with economic means seek private mental health care services that require private health insurance or out of pocket payments [50]. Unavailable and inadequate services disproportionately impact racialized youth [11]. In a scoping review on barriers and facilitators to accessing mental healthcare in Canada for Black youth, the authors found that financial challenges were frequently identified as a barrier to mental health care for Black youth [11]. Affordability affects families supporting youth with mental health concerns. Lin et al. [51] examined the costs associated with caregiving and found that caregivers experience financial strain and need to pay out of pocket costs for medications, as well as travel, treatment, and time lost from employment to attend appointments which further divides access to mental healthcare along socioeconomic lines.

Another structural barrier to mental healthcare access identified in our study is appropriateness and adequacy of services. Services did not fit the needs of youth and families for several reasons including a lack of specialized services, lack of family focused approaches to care, format of service delivery and poor-quality mental health care. In a study on health care needs in Ontario, Nelson and Park [4] found that young people aged 15–24 are eight times more likely to have unmet needs than adults 65 and over. The needs of Ontario caregivers are also unmet, and Miller et al. [16] found that there is an inadequate system of care “that may poorly understand and underuse the role of the caregiver in supporting interventions and optimal outcomes for youth and young adults dealing with a mental health issue.” ([16], p315) Parents of transition aged youth (aged 16–24) with mental health concerns have greater challenges than parents of younger children as they are trying to access services in an adult mental health care system that does not systematically involve families in their youth’s mental health treatment [8, 16].

Malla et al. [52] argue that specialized services continue being divided in separate silos despite the high rates of concurrent disorders in youth. Youth in need of specialized mental health or neurodevelopmental services experienced challenges finding appropriate services pre-pandemic, but this has amplified during the pandemic. Since the start of the pandemic, there have been numerous studies showing service disruptions [53] and inadequate and inappropriate services for specialized areas such as eating disorders [54], attention deficit hyperactivity disorder, and autism [44]. Hawke and colleagues [55] found that transgender and gender diverse youth had service disruptions to physical health services such as gender-affirming therapies, as well as interruptions to mental health and substance use services. Consistent with other studies, we also found that virtual care is not an appropriate or adequate form of service delivery for *all* youth [53].

Another structural barrier to mental health care access was acceptability of services and service providers. Acceptability includes stigma [4], racism, lack of culturally responsive services [11], as well as negative experiences with service providers such as lack of empathy and feeling judged [16, 49, 55]. Nelson and Park [4] found that a high rate of individuals with unmet healthcare needs identify acceptability as the most frequently reported structural barrier. Studies shown have also found that caregivers feel devalued and perceive some of the services as hostile in response to their request to be involved in their youth’s care [8, 16]. A review on barriers and facilitators to mental healthcare for Black youth in Canada emphasized the Eurocentric nature of mental health services and the importance of culturally responsive services and affirming care for Black youth and their families [11].

A final structural barrier identified by our participants is linked with policies, procedures, and laws which is an area that is not encompassed adequately by the Levesque et al. [18] conceptual framework on access to healthcare. Understanding how policies influence access to care from the perspectives of parents, youth, and service providers is an important contribution of this study. This contribution connects to the essence of structural barriers defined in the introduction as barriers stemming from “institutional policies and procedures” ([22], p1372) and “systematically hindering access for certain groups of people.” ([21], p215) Our findings underlined that policies and laws such as consent, choice, autonomy, and confidentiality influence practices and procedures that exclude families and caregivers. McNeil [56] argues that families experience structural discrimination through policies that “intentionally or unintentionally restrict the opportunities of people with mental health issues and their families” ([56], p57]). The author notes that the largest barrier to family-centred practice is inequitable distribution of power. The unpaid contributions of parents and caregivers are inadequately recognized and not supported by the mental health care system. Williams [57] also underlines the lack of recognition of *caregiving families* particularly in individualistic cultures in countries such as Canada. There are institutional policies and practices “in which families are expected to promote the health and wellbeing of family members while the state withholds and withdraws support” ([57], p75). The author adds that “families are living under conditions of siege” ([57], p75) as they are unrecognized, isolated, overburdened, and unsupported. It is important to note that this theme was largely informed by the experiences and perspectives of parents and service providers. However, youth also described the need to have more parent involvement in their mental health treatment and attributed the lack of involvement to not having culturally responsive services. It is important to understand that the absence or limited presence of culturally responsive services which impeded families from being involved in their youth’s treatment are stemming from systemic and structural barriers including inadequate training of mental health care professionals on working with youth and families, institutional policies on engaging families using culturally responsive interventions, and a Eurocentric mental health care treatment system design [11].

Participants in this study described the benefits of navigation services to help reduce barriers impeding access to youth mental health care. Navigation services promote individual and family-focused approaches to care and support youth and families across the care trajectory [58]. Navigators for mental health needs can help match youth and families’ needs to providers with the most appropriate and specialized expertise [59, 60]. Parents also emphasized the value of peer support services which were available when professional services were not. Peer support services are evidence informed and aligned with principles of recovery-oriented care [61]. Peer support workers use their lived experiences to support others having difficulty and this can help reduce stigma and instill hope [62]. A study on navigating youth mental health care in Ontario during the pandemic found that navigators experienced challenges helping families because many services were unavailable. [8] This is consistent with a study examining the experiences of caregivers supporting youth in seeking mental health care during the pandemic. Parents in this study described the lack of available services and expressed frustration, despair, isolation, and a “sense of being left behind by the system” ([63], p1).

### Strengths and limitations

Our study’s key strength is its exploration of youth mental health care, a critical area in crisis which was explored through the perspectives of parents, youth, and service providers. Trustworthiness was strengthened through collaboration between the university and community partner, as well as researcher and data triangulation. The research team included diverse interdisciplinary team members and advisory committee members with professional and lived experiences which provided greater depth and breadth to our understanding of youth access to mental health care. The research team combined professional and lived expertise to reduce researcher bias and co-generate knowledge on access to youth mental health care. Other strengths included prolonged engagement by the research team with youth and families coping with mental health and addictions concerns. The study strengthened transferability by using thick description to contextualize access to mental health care with an in-depth focus on different aspects related to access from different stakeholders.

The study also had a few limitations including a small number of youth participants. Our recruitment was limited to youth and families receiving services from the Family Navigation Project and at the time of recruitment, there were significantly more parents initiating navigation services than youth which limited our pool of youth for this study. We achieved saturation with each stakeholder group but would recommend further research to have a deeper understanding of youth and parent perspectives on these themes, especially now that restrictive pandemic measures are no longer in place. We would also recommend that future research aims to have greater representation of low barrier, community-based agencies. Another limitation is the overrepresentation of female participants across both service users and service providers. Most parents were mothers, most service providers identified as female, and all youth participants identified as female. However, almost all the mothers in our study were supporting sons with mental health concerns. This would be an important area for future research to better understand how sex and gender influence treatment seeking and access to youth mental health care, as well as parent or caregiver involvement in a youth’s mental health treatment.

## Conclusion

This study has important implications for mental health service provisions and policies. Even before the pandemic, there were calls to address a national mental health crisis in Canadian youth. Our findings showed that while structural barriers to youth mental health care access were present before the pandemic, the barriers increased significantly during the pandemic. There were increased mental health and addiction concerns in youth during the pandemic with decreased services and inadequate mental health care. The lack of services, increased youth needs, and lack of family involvement by service providers has placed tremendous burden on parents. Having Canadian youth in distress for this long shows that this is not a priority for the government, and policies implemented during the pandemic did not make children and youth a priority and they have widened mental health disparities and inequities in access to care. A recent report by Follwell et al. [64] described an initiative by the Mental Health Commission of Canada and HealthCare*CAN* to develop a framework that identifies essential dimensions of quality mental healthcare that meets the needs of individuals and communities. One of the dimensions included in this framework is accessible care that is timely, equitable, and promotes prevention and early intervention. It is critical that we advocate for a pandemic recovery strategy that will prioritize Canadian children and youth and will engage parents and youth in the development of policies that reduce barriers and increase access to mental health care for Canadian youth and families.

## Data Availability

Not applicable.

## Footnotes

1 AOP refers to anti-oppressive practice.

## References

[1] Pearson C, Janz T, Ali J. Health at a glance: mental and substance use disorders in Canada. Ottawa: Statistics Canada; 2013 Sep. 8 p. Cat. No.: 82-624-X. https://www150.statcan.gc.ca/n1/en/pub/82-624-x/2013001/article/11855-eng.pdf?st=RFXXQ31y. Accessed 8 Sep 2013.

[2] Wiens K, Bhattarai A, Pedram P, Dores A, Williams J, Bulloch A (2020). A growing need for youth mental health services in Canada: examining trends in youth mental health from 2011 to 2018. Epidemiol Psychiatr Sci.

[3] Boak A, Elton-Marshall T, Hamilton HA. The well-being of Ontario students: findings from the 2021 Ontario Student Drug Use and Health Survey (OSDUHS). Toronto: Centre for Addiction and Mental Health. 2022. https://www.camh.ca/-/media/files/pdf---osduhs/2021-osduhs-report-summary-pdf.pdf. Accessed 15 April 2022.

[4] Nelson CH, Park J (2006). The nature and correlates of unmet health care needs in Ontario. Canada Soc Sci Med.

[5] Sukhera J, Lynch J, Wardrop N, Miller K (2017). Real-time needs, real-time care: creating adaptive systems of community-based care for emerging adults. Can J Commun Ment Health.

[6] Waddell C, McEwan K, Shepherd CA, Offord DR, Hua JM (2005). A public health strategy to improve the mental health of Canadian children. Can J Psychiatry.

[7] Singh SP, Paul M, Ford T, Kramer T, Weaver T, McLaren S (2010). Process, outcome, and experience of transition from child to adult mental healthcare: multiperspective study. Br J Psychiatry.

[8] Kourgiantakis T, Markoulakis R, Hussain A, Lee E, Ashcroft R, Williams CC, Lau C, Goldstein A, Kodeeswaran S, Levitt A (2022). Navigating inequities in the delivery of youth mental health care during the COVID-19 pandemic: perspectives of youth, families, and service providers. Can J Public Health.

[9] Kowalewski K, McLennan JD, McGrath PJ (2011). A preliminary investigation of wait times for child and adolescent mental health services in Canada. J Can Acad Child Adolesc Psychiatry.

[10] Cairney J, Gandhi S, Guttmann A, Iron K, Khan S, Kurdyak P, et al. The mental health of children and youth in Ontario: a baseline scorecard. Toronto: Institute for Clinical Evaluative Sciences. 2015. 205 p. https://www.ices.on.ca/flip-publication/MHASEF_Report_2015/files/assets/common/downloads/publication.pdf. Accessed 3 Mar 2022.

[11] Fante-Coleman T, Jackson-Best F (2020). Barriers and facilitators to accessing mental healthcare in Canada for black youth: a scoping review. Adolesc Res Rev.

[12] Mental Health Commission of Canada. Taking the next step forward: building a responsive mental health and addictions system for emerging adults. Ottawa: Mental Health Commission of Canada. 2015. 107 p. https://www.mentalhealthcommission.ca/wp-content/uploads/drupal/Taking%252520the%252520Next%252520Step%252520Forward_0.pdf. Accessed 23 Jan 2022.

[13] Craig SL, Eaton AD, Leung VW, Iacono G, Pang N, Dillon F (2021). Efficacy of affirmative cognitive behavioural group therapy for sexual and gender minority adolescents and young adults in community settings in Ontario. Canada BMC Psychol.

[14] Schraeder KE, Reid GJ (2015). Why wait? The effect of wait-times on subsequent help-seeking among families looking for children’s mental health services. J Abnorm Child Psychol.

[15] Markoulakis R, Chan S, Levitt A (2020). The needs and service preferences of caregivers of youth with mental health and/or addictions concerns. BMC Psychiatry.

[16] Miller K, Sukhera J, Lynch J, Wardrop N (2017). Voices unheard: exploring the caregiver experience for caregivers of emerging adults with mental illness. Fam Soc.

[17] Cu A, Meister S, Lefebvre B, Ridde V (2021). Assessing healthcare access using the Levesque’s conceptual framework– a scoping review. Int J Equity Health.

[18] Levesque JF, Harris MF, Russell G (2013). Patient-centred access to health care: conceptualising access at the interface of health systems and populations. Int J Equity Health.

[19] Reardon T, Havery K, Baranowska M, O’Brien D, Smith L, Creswell C (2017). What do parents perceive are the barriers and facilitators to accessing psychological treatment for mental health problems in children and adolescents? A systematic review of qualitative and quantitative studies. Eur Child Adolesc Psychiatry.

[20] Priester MA, Browne T, Iachini A, Clone S, DeHart D, Seay KD (2016). Treatment access barriers and disparities among individuals with co-occurring mental health and substance use disorders: an integrative literature review. J Subst Abuse Treat.

[21] Kalmuss D, Fennelly K (1990). Barriers to prenatal care among low-income women in New York City. Fam Plann Perspect..

[22] Carbonell A, Navarro-Pérez JJ, Mestre MV (2020). Challenges and barriers in mental healthcare systems and their impact on the family: a systematic integrative review. Health Soc Care Community.

[23] Government of Canada. Canada Health Act. Ottawa: Department of Justice. 1984. https://laws-lois.justice.gc.ca/eng/acts/c-6/fulltext.html. Accessed 12 Dec 2017.

[24] Hutchison B, Levesque JF, Strumpf E, Coyle N (2011). Primary health care in Canada: systems in motion. Milbank.

[25] Statistics Canada. Population estimates, quarterly. Ottawa: Statistics Canada. Table 17-10-0009-01, Population estimates, quarterly. https://www150.statcan.gc.ca/t1/tbl1/en/tv.action?pid=1710000901. Accessed 17 Mar 2022.

[26] Ashcroft R (2015). Ontario’s Family Health Teams: politics within the model. Can Soc Work Rev.

[27] Ashcroft R, Menear M, Silveira J, Dahrouge S, Emode M, Booton J, McKenzie K (2021). Inequities in the delivery of mental health care: a grounded theory study of the policy context of primary care. Int J Equity Health.

[28] Miller K, Hameed S, Sukhera J (2020). Lost together: experiences of family physicians with emerging adult mental health. Can Fam Phys.

[29] Sandelowski M (2010). What's in a name? qualitative description revisited. Res Nurs Health.

[30] Jull JE, Davidson L, Dungan R, Nguyen T, Woodward KP, Graham ID (2019). A review and synthesis of frameworks for engagement in health research to identify concepts of knowledge user engagement. BMC Med Res Methodol.

[31] Piat M, Sabetti J (2009). The development of a recovery-oriented mental health system in Canada: what the experience of commonwealth countries tells us. Can J Commun Ment Health.

[32] Markoulakis R, Weingust S, Foot J, Levitt A (2016). The family navigation project: an innovation in working with families to match mental health services with their youth’s needs. Can J Commun Ment Health.

[33] Government of Ontario. Ontario enacts declaration of emergency to protect the public. Toronto: Office of the Premier. https://news.ontario.ca/en/release/56356/ontario-enacts-declaration-of-emergency-to-protect-the-public. Accessed 17 Mar 2020.

[34] Gadermann AC, Thomson KC, Richardson CG, Gagné M, McAuliffe C, Hirani S (2021). Examining the impacts of the COVID-19 pandemic on family mental health in Canada: findings from a national cross-sectional study. BMJ Open.

[35] Wu T, Jia X, Shi H, Niu J, Yin X, Xie J (2021). Prevalence of mental health problems during the COVID-19 pandemic: a systematic review and meta-analysis. J Affect Disord.

[36] Tseng V. Health footprint of pandemic [Image on internet]. 2020. https://twitter.com/VectorSting/status/1244671755781898241. Accessed 3 April 2022

[37] Patton MQ (2002). Qualitative research & evaluation methods.

[38] DiCicco-Bloom B, Crabtree BF (2006). The qualitative research interview. Med Educ.

[39] Braun V, Clarke V (2006). Using thematic analysis in psychology. Qual Res Psychol.

[40] Lincoln YS, Guba EG (1985). Naturalistic inquiry.

[41] Cleverley K, Rowland E, Bennett K, Jeffs L, Gore D (2020). Identifying core components and indicators of successful transitions from child to adult mental health services: a scoping review. Eur Child Adolesc Psychiatry.

[42] Saunders NR, Kurdyak P, Stukel TA, Strauss R, Fu L, Guan J (2022). Utilization of physician-based mental health care services among children and adolescents before and during the COVID-19 pandemic in Ontario Canada. JAMA Pediatr.

[43] Vaillancourt T, Szatmari P, Georgiades K, Krygsman A (2021). The impact of COVID-19 on the mental health of Canadian children and youth. Facets.

[44] Cost KT, Crosbie J, Anagnostou E, Birken CS, Charach A, Monga S (2021). Mostly worse, occasionally better: impact of COVID-19 pandemic on the mental health of Canadian children and adolescents. Eur Child Adolesc Psychiatry.

[45] Robillard R, Daros AR, Phillips JL, Porteous M, Saad M, Pennestri MH (2021). Emerging new psychiatric symptoms and the worsening of pre-existing mental disorders during the COVID-19 pandemic: a Canadian multisite study: Nouveaux symptômes psychiatriques émergents et détérioration des troubles mentaux préexistants durant la pandémie de la COVID-19: une étude canadienne multisite. Can J Psychiatry.

[46] Stewart SL, Vasudeva AS, Van Dyke JN, Poss JW (2021). Child and youth mental health needs and service utilization during COVID-19. Traumatology.

[47] Fegert JM, Vitiello B, Plener PL, Clemens V (2020). Challenges and burden of the coronavirus 2019 (COVID-19) pandemic for child and adolescent mental health: a narrative review to highlight clinical and research needs in the acute phase and the long return to normality. Child Adolesc Psychiatry Ment Health.

[48] Hawke LD, Barbic SP, Voineskos A, Szatmari P, Cleverley K, Hayes E (2020). Impacts of COVID-19 on youth mental health, substance use, and well-being: a rapid survey of clinical and community samples: Répercussions de la COVID-19 sur la santé mentale, l’utilisation de substances et le bien-être des adolescents: un sondage rapide d’échantillons cliniques et communautaires. Can J Psychiatry.

[49] Stunden C, Zasada J, VanHeerwaarden N, Hollenberg E, Abi-Jaoudé A, Chaim G (2020). Help-seeking behaviors of transition-aged youth for mental health concerns: qualitative study. J Med Internet Res..

[50] Ronis ST, Slaunwhite AK, Malcom KE (2017). Comparing strategies for providing child and youth mental health care services in Canada, the United States, and the Netherlands. Adm Policy Ment Health.

[51] Lin E, Durbin J, Guerriere D, Volpe T, Selick A, Kennedy J (2018). Assessing care-giving demands, resources and costs of family/friend caregivers for persons with mental health disorders: a scoping review. Health Soc Care Community.

[52] Malla A, Shah J, Iyer S, Boksa P, Joober R, Andersson N (2018). Youth mental health should be a top priority for health care in Canada. Can J Psychiatry.

[53] Hawke LD, Sheikhan NY, MacCon K, Henderson J (2021). Going virtual: youth attitudes toward and experiences of virtual mental health and substance use services during the COVID-19 pandemic. BMC Health Serv Res.

[54] Couturier J, Pellegrini D, Miller C (2021). The COVID-19 pandemic and eating disorders in children, adolescents, and emerging adults: virtual care recommendations from the Canadian consensus panel during COVID-19 and beyond. J Eat Disord.

[55] Hawke LD, Hayes E, Darnay K, Henderson J (2021). Mental health among transgender and gender diverse youth: an exploration of effects during the COVID-19 pandemic. Psychol Sex Orientat Gend Divers.

[56] McNeil S (2013). Understanding family-centered care in the mental health system: perspectives from family members caring for relatives with mental health issues. Soc Work Ment Health.

[57] Williams C (2018). Caregiving under siege: an argument for critical social work practice with families. critical social work: past, present, and future. Can Soc Work.

[58] Markoulakis R, Luke A, Reid A, Mehra K, Levitt A, Doucet S (2021). Proceedings of the inaugural Canadian healthcare navigation conference: a forum for sharing innovations and best practices in navigation services. BMC Proc.

[59] Bieling PJ, Madsen V, Zipursky RB (2013). A ‘navigator’ model in emerging mental illness?. Early Interv Psychiatry.

[60] Knesek G, Hemphill T (2020). Mental health navigation–—a model. Health Promot Int.

[61] Mutschler C, Bellamy C, Davidson L, Lichtenstein S, Kidd S (2021). Implementation of peer support in mental health services: a systematic review of the literature. Psychol Serv.

[62] Markoulakis R, Turner M, Wicik K, Weingust S, Dobbin K, Levitt A (2018). Exploring peer support needs of caregivers for youth with mental illness or addictions concerns in family navigation services. Community Ment Health J.

[63] Markoulakis R, Da Silva A, Kodeeswaran S, Levitt A (2022). Youth mental health and/or addiction concerns and service needs during the COVID-19 pandemic: a qualitative exploration of caregiver experiences and perspectives. Child Adolesc Psychiatry Ment Health.

[64] Follwell EJ, Chunduri S, Samuelson-Kiraly C, Watters N, Mitchell JI (2021). The quality mental health care network: a roadmap to improving quality mental healthcare in Canada. Healthc Manage Forum.

